# Supplementary material for: Social Support Initiatives That Facilitate Exercise Participation in Community Gyms for People with Disability: A Scoping Review
Source: Int J Environ Res Public Health. 2022 Dec 30;20(1):699. doi: 10.3390/ijerph20010699 (PMC9819822; doi:10.3390/ijerph20010699)
Supplement: Supplementary file 1 [file ijerph-20-00699-s001.zip › ijerph-2033542-supplementary.docx]

**Supplementary Files:**

Table S1. Social support strategies in community gyms – Supervision

| Authors (year) | **Who provided supervision?** | **Ratio trainer:**  **participant** | **Disability experience and training of those providing supervision** | **Additional information** |
| --- | --- | --- | --- | --- |
| *Adolescents and young adults (15-30 years) n=7* | | | | |
| Pett et al. (2013) [38] | Health and exercise personnel  Volunteers |  | Trained exercise and health care personnel from the recreation centre | Health and exercise personnel monitored closely participant safety. Volunteers were recruited from a local university and the community. |
| Shields et al. (2008) [33] | Fitness trainers |  | Not described |  |
| Shields et al. (2020) [32] | Physiotherapists | 1:2 or 1:3 | Usual practice was in the areas of paediatrics, neurology or musculoskeletal physiotherapy.  Received a training manual. | Training manual included details of the principles of progressive resistance training, an overview of program content and tips for working with people with Prader-Willi syndrome. |
| Stanish & Temple (2012^b^) ^+#^ [57]  Temple & Stanish (2011) ^#^ [47]  Stanish & Temple (2012^a^) ^+^ [55] | Certified fitness trainers | 1:1 | Two 90-min training sessions | Training included information on ID, strategies for communication, social interaction, mutual respect, motivation and positive feedback, basic principles of safe exercise training. Supervised and co-ordinated sessions; provided instructional support to ensure safe technique. Monitored exercise plans and adjusted as participants demonstrated gains. |
| Taylor et al. (2013) ^#^ [36]  Bania et al (2016) ^#^ [58] | Physiotherapist |  | Not described |  |
| Zanudin et al. (2021) [48] | Fitness instructor | 1:1 or 1:2 | Attended at least 3 sessions supervised by a physiotherapist | Available to assist and provide instruction to participants at any session. |
| *Adults (> 30 years) n=23* | | | | |
| Allen et al. (2004) ^#^ [50]  Taylor et al. (2004) ^#^ [45] | Exercise physiologist  Fitness instructor  Support staff |  | Support staff from supported accommodation had experience working with people with physical disabilities. | The exercise physiologist led each exercise session for a group of 5-6 participants, assisted by a registered fitness instructor and 2 support staff. |
| Carter et al. (2004) [54] | Certified Therapeutic Recreation Specialist  Graduate assistant  Undergraduate therapeutic recreation students |  | Therapeutic Recreation Specialist and graduate assistant experienced in inclusive fitness programming.  Students received training and support from the specialist. | Student partners provided motivation, counted and recorded repetitions, monitored time and behaviour and completed warm-up and cool-down exercises with participants. |
| Corcos et al. (2013) [24] | ACE or ACSM certified personal trainer | 1:2 | Experience working with individuals with limited function. Training on the appropriate exercise program. | Training twice per week for 6 months followed by once per week for 18 months. Program coordinator contacted trainers every 2-3 months |
| Danoudis & Iansek (2021) [41] | Qualitied gym instructor | 1:1 (student) | 3-hour training session on topics related to PD delivered a physiotherapist experienced in movement disorders. Ongoing support was provided as required by physiotherapists. | Gym instructor was present at each session, however participants exercised independently alongside other participants.  They were responsible for monitoring safety and recording falls and adverse events. |
| Dodd et al. (2006) ^#^ [51]  Taylor et al. (2006) ^#^ [46] | Physiotherapist  Accredited fitness instructors | 1:1 | Physiotherapist was employed by the MS society | Physiotherapist led the program assisted by two fitness instructors |
| Dodd et al. (2011) [25] | Physiotherapist  Registered personal sport trainers |  | ‘Experienced trainers’ | Up to 12 participants supervised by up to 3 trainers (physiotherapist and personal trainers) |
| Duret et al. (2020) [42] | Adapted physical activity instructors | 1:3 | Skilled in training people with neurological disorders | Employed by a rehabilitation centre. After 6-month program, participants could continue to train with supervision of fitness centre employees.  Closer supervision was provided to new group participants while gaining independence to the level of the other group members. |
| Elsworth et al. (2011) ^#^ [26]  Winward et al. (2011) ^#^ [60] | Fitness trainers | 1:3 or 1:4 | On UK register of exercise professionals level 3 or above. Expertise in exercise for people with disability. Provided with information on exercise for people with neurological conditions and practice advice from a physiotherapist. | Other gym staff trained in disability awareness. |
| Fenton et al. (2021) [27] | Fitness instructors | 1:10 or 1:12 | Trained about exercise in rheumatoid arthritis from an exercise physiologist with experience in prescribing exercise for those with rheumatoid arthritis. | Were available to answer questions. Training included information about rheumatoid arthritis and identifying barriers to participation. Instructors also received 2x 1.5-hour sessions about SDT and promoting autonomy and support to participants. |
| Handlery et al. (2022) [49] | Physiotherapists Physiotherapy students |  | Physiotherapists and students were trained instructors for a group-based physical activity programme. | Instructors fostered a “fun” environment by using fun games and other motivational strategies and provided verbal education on the importance of aerobic activity and the American Heart Association’s guidelines for physical activity post stroke. |
| Hansen et al. (2018) [52] | Gym instructor (part 2) |  | Not described | The instructor guided and supervised the first gym session only. |
| Hassett et al. (2009) [14] | Personal trainer |  | Received written information about traumatic brain injury, the trial and the intervention. Trainers contacted by researchers at the start of the program to review exercises. | Personal trainer determined how to complete and progress the exercises. Contact with trainers once program commenced to answer any queries regarding the prescribed program. |
| Hoffman et al. (2010) [28] | Certified athletic trainer  Educational trainer |  | Educational trainer trained in social cognitive therapy and trans-theoretical stages of change model |  |
| Kovacic et al. (2020) [29] | *Group 1 -* Physiotherapist Physiotherapy students  *Group 2* - Fitness instructor | 1:1 | Physiotherapist specialist in neurodevelopmental physiotherapy | Group 1: Physiotherapists supervised once per week  Students supervised twice per week  Group 2: Fitness instructor once per week |
| Lampousi et al (2020) [30] | Personal trainers |  | ‘Experienced’ personal trainers had 2-3 group meetings to discuss intervention training practices. | Trainers were personal trainers employed by the fitness centres where the training took place. |
| Morris et al. (2009) 43] | Physiotherapist | *Group 1*  1:1 (student) | Not described |  |
| Obrusnikova et al (2019) ^+^ [56]  Obrusnikova et al. (2021^a^) ^+ ^^ [59]  Obrusnikova et al. (2021^b^) ^^^ [40] | Instructor / coach  Research assistant  (Both college students) | 1:1 | A supervisor (a study investigator) independently observed (directly or indirectly) 50% of sessions and checked against a checklist to ensure >90% fidelity. If <90%, the instructor was provided with feedback and practice opportunity. | Instructors/coach and research assistants were college students in health and behaviour science or exercise science. Training included strategies for working with adults with ID, information on confidentiality and procedures, the research protocol and instructional script. Instructors/coach were videoed to ensure procedural adherence. |
| Pau et al. (2018) [31] | Accredited strength and conditioning coaches  Physician | 1:1 | Not described | Physician was constantly present to assist coaches. |
| Ploughman et al. (2014) [44] | Physiotherapist  Exercise partners (volunteer university students) | 1:1 | Exercise partners received a  2-hour training session | Physiotherapist was responsible for exercise progression and equipment modifications.  Exercise partners training covered neurological conditions, precautions and safety. Exercise partners assigned when participant transitioned from circuit training to using standard gym equipment. 2:1 with participants with considerable balance, cognitive/behavioural concerns. |
| Poliakoff et al. (2013) 39] | Gym trainers | 2:4 or 2:3 | Previous experience working with people with Parkinson disease |  |
| Sims et al. (2009) [35] | Accredited fitness trainer | 1:1 | Not described | Fitness trainer logged attendance and any problems arising during the program. |
| Wallace et al. (2019) [37] | Fitness instructor |  | Face to face training with a physiotherapist and received a training manual. | Supervised participant’s exercise sessions weekly for 1 month and then every other week for 2 months. |

^#^ Separate papers reporting the same study; ^^^ Same study, 2021^a^ reports 3-week familiarisation phase, 2021^b^ reports full study 3 weeks familiarisation and 10-week intervention study; ^+^ Separate studies with similar methods

Table S2. Social support strategies in community gyms - Peer support

| Authors (year) | Who provided peer support? | Format of peer support | Additional information |  |
| --- | --- | --- | --- | --- |
| *Adolescents and young adults (15-30 years) n=9* | | | | |
| Pett et al. (2013) [38] | Peers with intellectual disability | Group exercise | Participants exercised in a supervised group of 11 or 12 |  |
| Shields et al. (2008) [33] | Peers with Down syndrome | Small group exercise | Participants exercised in supervised groups of 2-3 to allow for a close level of supervision. |  |
| Taylor et al. (2013) ^#^ [36]  Bania et al (2016) ^#^ [58] | Peer with cerebral palsy | Participants trained in pairs | Participants exercised either individually or in pairs under the supervision of a physiotherapist. |  |
| Stanish & Temple (2012^b^) ^# +^ [57]  Temple & Stanish (2011) ^#+^ [47] | Peer partner (local high school students)  Peers with intellectual disability | 1:1 or 1:2 within a larger group | Pairs/triads exercised at a set time each week under supervision of a fitness trainer. Eight (8) participants exercised 1:1 and 6 participants exercised alongside a peer with intellectual disability in addition to peer exercise partner (1:2). The peer-participant relationship was an equal and reciprocal partnership rather than ‘tutor-tutee’ and the peer partners exercised alongside their partner(s). |  |
| Stanish & Temple (2012^a^) ^+^ [55] | Peer mentor (local high school students)  Peers with intellectual disability | 1^st^ session per week 1:2  2^nd^ session per week 1:1 (intellectual disability only) | Pairs/triads exercised at a set time each week under supervision of a fitness trainer. First session of each week two participants with intellectual disability paired with a peer mentor - the triad exercised together and offered reciprocal support as they rotated through the stations. The peer mentor acted as a ‘‘workout buddy’’ and assisted his or her two partners with intellectual disability as needed. The second session of each week the peer partners with intellectual disability exercised together without the peer mentor. |  |
| Shields & Taylor. (2010) ^+^ [17] Shields et al. (2013) ^+^ [18] | Peer mentor (volunteer or paid physiotherapy students) | 1:1 | Mentors exercised with the participants. |  |
| Shields et al. (2019) [53] | Peer mentor (volunteer university students enrolled in physiotherapy or exercise physiology) | 1:1 | Mentors exercised with the participants. |  |
| Shields et al. (2021) ^#^ [34]  McKenzie et al. (2022) ^#^ [15] | Peer mentor (volunteer university students enrolled in a health discipline) | 1:1 | Mentors exercised with the participants.  Mentors of participants with more complex needs did not exercise to focus on providing support. |  |
| *Adults (> 30 years) n=12* | | | | |
| Allen et al. (2004) ^#^ [50]  Taylor et al. (2004) ^#^ [45] | Peers with cerebral palsy | Small group exercise | Participants exercised in a supervised group of 5-6. |  |
| Danoudis and Iansek (2021) [41] | Peers with Parkinson disease  Family member | Group exercise | Participants undertook a personalised exercise program alongside other people with Parkinson disease in a supervised group.  Family members could take out a gym membership and exercise alongside their partner with Parkinson disease. |  |
| Dodd et al. (2006) ^#^ [51]  Taylor et al. (2006) ^#^ [46] | Peers with multiple sclerosis | Small group exercise | Participants exercise in a supervised group of 3. |  |
| Dodd et al. (2011) [25] | Peers with multiple sclerosis | Small group exercise within a larger group | Up to 12 participants were split into smaller supervised groups of 3-4 and exercised concurrently in the gym. |  |
| Duret et al. (2020) [42] | Peers with neurological disorders | Group exercise | Participants with similar levels of ability and independence exercised in a supervised group of 10 to 12. |  |
| Handlery et al. (2022) [49] | Peers who were stroke survivors  Family member | Group exercise | 15 participants and 6 carers exercised together in a supervised setting for the first 8 weeks. Family member could exercise alongside participant. |  |
| Pau et al. (2018) [31] | Peers with multiple sclerosis | Small group exercise | Participants exercised in a supervised group of 3 or 4. Group instructor to participant ratio was 2:4 or 2:3 |  |
| Poliakoff et al. (2013) [39] | Peers with Parkinson disease | Group exercise | Participants completed an individualised program in a supervised group of 15 participants. |  |
| Sims et al. (2009) [35] | Peers who had survived stroke | Small group exercise | Participants exercised in small, supervised groups. |  |
| Kovacic et al. (2020) [29] | *Group 1:* Peers with intellectual disability and physiotherapy students  *Group 2:* Peers with intellectual disability | *Group 1*: group session x 1 and 1:1 session x 2 per week  *Group 2:* group exercise | *Group 1:* Participants engaged in one supervised group session per week with a physiotherapist and two 1:1 session with a physiotherapy student.  *Group 2:* Participants exercised in a larger group once per week |  |
| Ploughman et al. (2014) [44] | Peers with neurodisability  Exercise partners (volunteer university students) | Group exercise  1:1 | Participants exercised in a supervised group of 9 to 10 in a smaller exercise area within the gym facility before transitioning 1:1 with an exercise partner to the main gym between weeks 2 to 6 of the 10-week program. Extra support (2:1 assistance) was provided for participants with considerable balance, cognitive or behavioural concerns. |  |
| Carter et al. (2004) [54] | Undergraduate therapeutic recreation students | 1:1 | Student partners counted and recorded repetitions, monitored time and behaviour and completed warm-up and cool-down exercises with participants. |  |

^#^ Separate papers reporting the same study; ^+^ Separate studies with similar methods

Table S3. Specialist supervision as a social support strategy in community gyms

| Authors (year) | Who provided the support | To whom was the support provided | Additional information | |
| --- | --- | --- | --- | --- |
| *Adolescents and young adults (15-30 years) n=5* | | | | |
| Shields & Taylor. (2010) ^+^ [17]  Shields et al. (2013) ^+^ [18] | Physiotherapist | Peer mentors | Mentor training included background information on Down syndrome, strategies for working with young people with Down syndrome including motivational, problem solving and teaching strategies, information about progressive resistance exercise such as progression and practical instruction in use of gym equipment.  Mentors had remote supervision every 2-3 weeks with the research team to review program and discuss any concerns. | |
| Shields et al. (2019) [53] | Experienced paediatric physiotherapist | Peer mentors | Mentor training included background information on disability, strategies for working with young people with disability including motivational and teaching strategies, information about exercise and practical instruction on how to implement the program at the gym.  Mentors had remote supervision every 2 weeks with the research coordinator to review program and discuss any concerns either via telephone, email or web-based exercise application. | |
| Shields et al. (2021) ^#^ [34]  McKenzie et al. (2022) ^#^ [15] | Exercise physiologists and physiotherapists experienced in paediatrics/disability | Peer mentors  Participants with disability – all  Participants with complex disability needs and their peer mentor | Mentor training and remote supervision per Shields et al. (2019).  Mentors for participants with complex disability received additional tailored training.  At the initial consultation the exercise program was tailored to the participants own goals, preferences and impairments.  Extra support for participants with complex disability included weekly monitoring and a physiotherapist attended the first gym session. | |
| Zanudin et al. (2021) [48] | Physiotherapist experienced in paediatrics and exercise referrals to leisure centres. | Fitness instructors  Participants with cerebral palsy | Attended 1st session and a further 2-4 visits during the first 12 weeks to monitor and adapt exercises to the ability of the individual participant. Fitness instructors attended also. | |
|  |  |  |  | |
| *Adults (> 30 years) n=14* | | | |  |
| Collett et al. (2016) [54] | Specialist exercise practitioner qualified in exercise for long term neurological conditions (level 4 qualification) or a physiotherapist | Participants with Parkinson disease | Initial orientation session then monthly visits. Exercise professional/physiotherapist could be contacted for further advice and information if required. | |
| Danoudis and Iansek (2021) [41] | Physiotherapists experienced in movement disorders | Participants with Parkinson disease  Gym instructors | Individual program prescribed by physiotherapist.  Physiotherapist delivered a 3-hour training session for gym instructors about Parkinson disease and working with people with Parkinson disease. Physiotherapists provided ongoing support and education to gym instructors as required | |
| Elsworth et al. (2011) ^#^ [26]  Winward et al. (2011) ^#^ [60] | Physiotherapist with expertise in long-term neurological conditions and exercise prescription | Participants with neurological conditions  Fitness trainers | Provided practical advice and support to participants at initial induction and then as required.  Physiotherapist provided Information on neurological conditions and safe exercise for these conditions to the trainers and provided support as required throughout the program. | |
| Fenton et al. (2021) [27] | Exercise physiologist with experience in developing exercise programs for people with rheumatoid arthritis. | Fitness instructors | Provided training about exercise in rheumatoid arthritis, including information about rheumatoid arthritis, developing a safe exercise program for people with rheumatoid arthritis and identifying physical and psychological barriers to participation ^65^. | |
| Handlery et al. (2022) [49] | NExT instructors who were physical therapists | Participants with stroke | Supported to transition from the group program to independent use of the gyms and phone calls to remind participants that this support was offered. | |
| Hansen et al. (2018) [52] | Occupational therapist | Participants with glioma who had received OT in Part 1 | Participants who received OT in Part 1 continued to receive a weekly 20-minute guided OT intervention in Part 2 | |
| Hassett et al. (2009) [14] | Physiotherapist | Personal trainers | Trainers were provided with written information about traumatic brain injury, the study and the intervention.  Trainers contacted once the program commenced to review the prescribed program. | |
| Hoffman et al. (2010) [28] | Educational trainer trained in social cognitive therapy and transtheoretical stages of change model | Participants with traumatic brain injury | Educational trainer provided motivational counselling which included identifying and resolving barriers to regular exercise. | |
| Kovacic et al. (2020) [29] | Physiotherapist (specialist in neurodevelopmental physiotherapy) | Participants with disability (Group 1) | Participants exercised in a group under the guidance of a physiotherapist once per week. | |
| Obrusnikova et al (2019) ^+^ [56] Obrusnikova et al. (2021^a^) ^+#^ [59]  Obrusnikova et al. (2021^b^) ^+#^ [40] | The researchers | Instructors and research assistants (college students) | Training for the instructors and research assistants included strategies for working with adults with intellectual disability, research protocol, procedures, and instructional script; instructors were videoed to ensure procedural adherence and competence; all research assistants and instructors were directly supervised by one of the investigators; supervisors observed 50% of all sessions and checked against a fidelity checklist to ensure >90% fidelity - if below this, the instructor was provided with feedback and opportunity to practice. | |
| Pau et al. (2018) [31] | Physician | Strength and conditioning coaches | Onsite support was provided to the coaches from the physician. | |
| Ploughman et al. (2014) [44] | Physiotherapists | Participants with neurological disorders  Exercise partner | Equipment was modified and exercise adapted as required. | |
| Wallace et al. (2019) [37] | Physiotherapist | Fitness instructors  Participants with neuromuscular disease | Completed face-to-face training with fitness instructors.  Attended the gym at the beginning and midpoint of the program.  Conducted a telephone review every 2 weeks with participants.  Extra visits or telephone calls provided to the participant or fitness instructor where they identified a need for more support.  Monitored participant’s exercise diary during fortnightly phone-calls and mid-way gym visit | |

+ Separate studies with similar methods # Separate papers reporting the same study

Table S4. Orientation or familiarisation as a social support strategy in community gyms

| Authors (year) | Format | Number of sessions | Who completed the orientation? | Additional information |
| --- | --- | --- | --- | --- |
| *Adolescents and young adults (15-30 years) n=3* | | | | |
| Shields et al. (2021) ^#^ [34]  McKenzie (2022) ^#^ [15] | Initial consultation | 1 | Exercise professional | Completed at the gym and used for prescribing an exercise program tailored to the participants based on their goals, preferences and impairments. |
| Stanish & Temple (2012^b^) ^#^ [57]  Temple & Stanish (2011) ^#^ [47] | Familiarisation period | 4 | Fitness instructors | To acquaint participants to the gym, equipment and staff, teach safety guidelines and correct technique, review gym etiquette, and teach how to record exercise program. |
| Zanudin et al. (2021) [48] | Familiarisation session | 1 | Physiotherapist | To adapt exercises to the ability of the individual participant. |
| *Adults (> 30 years) n=12* | | | | |
| Allen et al. (2004) ^#^ [50]  Taylor et al. (2004) ^#^ [45] | Introductory program | 4 | Exercise professional | Participants attended once a week for 4 weeks.  Week 1 participants were introduced to other group members and given opportunity to get used to program staff and venue. Weeks 2 to 4 participants learnt how to do the exercises with minimal resistance |
| Carter et al. (2004) [54] | Pre-program sessions | 2 | Graduate assistant | Sessions held in the week prior to the program.  Orientation to the venue - fitness room, gym and restrooms and how to navigate the building. Shown how to use gym equipment safely. Observations were made about the level of assistance needed e.g. ability to use the equipment safely (mounting/dismounting); understanding and adherence to the exercise routine; ability to count repetitions and compliance to directions. Individual adaptations made. |
| Collett et al. (2016) [23] | Initial gym session | 1 | Clinical exercise professional | Participants orientated to the environment, equipment, exercise program; ensured participants were competent and safe following the program. |
| Danoudis & Iansek (2021) [41] | First two sessions | 2 | Physiotherapist | Individualised program prescribed. Familiarisation with gym and equipment to ensure correct and safe use. |
| Dodd et al. (2006) ^#^ [51]  Taylor et al. (2006) ^#^ [46] | Familiarisation visits | 3 | Physiotherapist and fitness instructors | Attended over 4 weeks. Participants met other group members and program staff and learnt how to do the exercises with minimal resistance. |
| Elsworth et al. (2011) ^#^ [26]  Winward et al. (2011) ^#^ [60] | Initial induction session | 1 | Physiotherapist | Practical advice and support to participants were provided at this session. |
| Fenton et al. (2021) [27] | Gym induction | At least 1 | Gym instructors | Sessions were completed until the participant felt comfortable in the gym. |
| Handlery et al. (2022) [49] | Orientation session | 1 | Instructors | Written education on how to exercise safely was provided. |
| Hansen et al. (2018) [52] | Gym induction | 1 | Gym instructor | During their first gym session, participants were guided and supervised. |
| Morris et al. (2009) [43] | Familiarisation phase | 8 | Physiotherapist | An 8-week twice a week baseline phase prior to the program. An extra session at the end of baseline determined 1RM for each of the 8 exercises included in the training program and exercises were modified to each participant’s ability. |
| Obrusnikova et al (2019) ^1+^ [56] Obrusnikova et al. (2021^a^) ^2+^^ [59]  Obrusnikova et al. (2021^b^) ^2^^ [40] | Baseline sessions  Orientation sessions  Familiarisation period (2021^b^) | ^1^5-15 for baseline sessions  ^2^1-2 orientation sessions  3-week familiarisation period | Instructor/ coach | ^1.^ Baseline orientation session to instruct on the iPad operation and the video assisted schedule (VAS) app (First Then Visual Schedule (FTVS) app). Participants were familiarised with the exercise machines and the exercises. FTVS provided a video step-by-step breakdown of how to complete each exercise. These sessions continued until the participant displayed a stable or descending trend in requiring prompts (system of least to most prompting) to complete the exercises  ^2.^ In orientation sessions participants were shown how to use the 4 exercise machines and each exercise.  3-week period used to familiarise participants to the exercise procedures, gym setting and promote mastery of the resistance training exercises and equipment. Videos/photos of a model completing the exercises, wooden model of the pin-loaded weight mechanism to practice with and live demonstration from coach. Training with the VAS and SLMP iPad. Exercises included 10 min warm-up and 1 set of 10-12 repetitions performed at submaximal intensity (50-60% 1-RM) only. |

^#^ Separate papers reporting the same study; ^^^ Same study, 2021^a^ reports 3-week familiarisation phase, 2021 ^b^ reports full study 3 weeks familiarisation and 10-week intervention study; ^+^ Separate studies with similar methods

Table S5. Education, organised social activities, technology, logistical, motivational supports used in community gyms

| Authors (year) | Who provided the support? | Details of the support |
| --- | --- | --- |
| EDUCATION n = 7 | | |
| Hoffman et al. (2010) [28] | Educational trainer | Each session, participants received 15 mins of education on an exercise related topic; topics covered included general benefits of exercise, clothing and footwear, injury prevention and safety, motivation and barriers to exercise, nutrition and hydration, common physical complaints during exercise, exercising in the community, pacing and cross-training, and relapse prevention. Handouts were provided after each session. |
| Elsworth et al. (2011) [26]  Winward et al. (2011) [60] | Physiotherapist | To support gym-based exercise participation a Physical Activity Support System (PASS) was used which included information based on the “expert patient model” about exercise participation. Education included current exercise guidance (5 aerobic and 2 strength sessions per week) and encouragement to attend regularly. |
| Kovacic et al. (2020) [29] | *Group 1:* physiotherapist  *Group 2:* fitness instructor and nutritionist | *Group 1*: education program included workshops on active and healthy ageing and included behaviour change principles for initiating and maintaining structured physical activity.  *Group 2:* wellness and healthy lifestyle information, including regular physical activity, healthy nutrition, and adequate hydration. |
| Lampousi et al (2020) [30] | Health educator | Three 1:1 meetings with health educator to promote better physical activity and dietary habits during the program. Use of the LogMyFood food photography app-based program to log food and meals. Social media sharing function enabled for the intervention group only. |
| Obrusnikova et al (2019) ^+^ [56]  Obrusnikova et al. (2021^a^) ^+#^ [59]  Obrusnikova et al. (2021^b^) ^+#^ [40] | Instructor or coach | A video assisted schedule (VAS) – First Then Visual Schedule (FTVS) application was delivered on an iPad. The FTVS provided a videoed step-by-step breakdown of how to complete each exercise. |
| Obrusnikova et al. (2021^b^) [40] | Coaches | Information provided about commonly used exercise terms and how to use the exercise equipment and undertake the exercises independently. Participants educated about setting an exercise plan, including exercise intensity, performing exercises independently and monitoring plan using OMNI scales of perceived exertion. |
| Handlery et al. (2022) [49] | Program staff | Verbal education on the benefits of exercise and the purpose of specific exercise modalities provided throughout the 8 weeks supervised program. Tip sheets on how to exercise safely, taking BP and contraindication to exercise and ways to increase physical activity including local places to undertake activity provided at the beginning of the program. |
| ORGANISED SOCIAL ACTIVITIES n = 4 | | |
| Allen et al. (2004) [50]  Taylor et al. (2004) [45] | Program staff (exercise physiologist, fitness trainer and disability trained support staff)  Peers with CP | Each exercise session was followed by a short period of social interaction. |
| Dodd et al. (2006) [51]  Taylor et al. (2006) [46] | Program staff (physiotherapist and two fitness instructors)  Peers with MS | At the end of each session there was a period of 30 minutes to cool down and socialise over light refreshments. |
| Dodd et al. (2011) [25] | Program staff including physiotherapist and two personal trainers  Peers with MS | At the end of each session there was a period of 30 minutes to cool down and socialise over light refreshments. |
| Handlery et al. (2022) [49] | Program staff  Peer participants  Family member | Two recreational group outings – one at a local park in which recreational sports were played, and one at a local restaurant. |
|  |  |  |
| LOGISTICAL SUPPORT n = 6 | | |
| Carter et al. (2004) [54] | Social service agency | The agency from which the participants were recruited covered the cost of transportation to the gym. |
| Shields et al. (2008) [33] | Day program staff | Transport to/from the gym was facilitated by the day programs. |
| Sims et al. (2009) [35] | Research program | Transport provided if required. |
| Elsworth et al. (2011) [26]  Winward et al. (2011) [60] | Research program staff  Gym staff | Transport logistics including how to find the gym, transport options (volunteer transport services, taxis or public transport) were provided in a handbook. Research staff provided additional advice on the best route and transport option if the participant required it.  Informal support provided by gym staff including meeting participants in the car park and escorting them to the gym, viewing it as part of their role in enabling that person to exercise. |
| Pett et al. (2013) [38] | Research program | Free shuttle service provided to and from the recreation centre. |
| Handlery et al. (2022) [49] | Program staff | Physiotherapists and students were available to provide support on request to facilitate transition to independent physical activity at the gym. Each participant received one phone call to remind them of the offered support. |
|  |  |  |
| MOTIVATIONAL STRATEGIES n = 5 | | |
| Hassett et al. (2009) [14] | Personal trainer | The trainer and participant set physical activity goals related to the participant’s training program (3 month goal) and return to regular physical activity (6 month goal). |
| Corcos et al. (2013) [24] | Study coordinator | If two sessions in a row were missed, participants were contacted to resolve issues affecting compliance and program adherence. |
| Kovacic et al. (2020) [29] | Special Olympic coaches | Special Olympic coaches were recruited with each athlete participant to participate in regular meetings with the participant, to motivate and to support the participant in the multicomponent programme they were involved in. |
| Lampousi et al (2020) [30] | Personal trainer | Each training session included 5-10 mins of motivational interviewing and feedback from the trainer.  Use of the LogMyFood food photography app-based program to log food and meals. Could be shared via social media. |
| Obrusnikova et al. (2021^b^) [40] | Coaches | The participants set SMAP goals with the coaches and used a goal-setting tracker that converted performance data into graphs that could be visualised on an iPad. |

# Separate papers reporting the same study; +Separate studies with similar methods
